# Prenatal genetic analysis of fetal aberrant right subclavian artery with or without additional ultrasound anomalies in a third level referral center

**DOI:** 10.1038/s41598-023-30598-9

**Published:** 2023-02-28

**Authors:** Huili Xue, Lin Zhang, Aili Yu, Min Lin, Qun Guo, Liangpu Xu, Hailong Huang

**Affiliations:** 1 Medical Genetic Diagnosis and Therapy Center, Fujian Key Laboratory for Prenatal Diagnosis and Birth Defect, Fujian Maternity and Child Health Hospital College of Clinical Medicine for Obstetrics & Gynecology and Pediatrics, Fujian Medical University, No. 18 Daoshan Road, Gulou District, Fuzhou City, 350001 Fujian Province China; 2 Fujian Medical University, No. 88 Jiaotong Road, Cangshan District, Fuzhou City, 350001 Fujian Province China; 3 Reproductive Medicine Center, Fujian Maternity and Child Health Hospital College of Clinical Medicine for Obstetrics & Gynecology and Pediatrics, Fujian Medical University, No. 18 Daoshan Road, Gulou District, Fuzhou City, 350001 Fujian Province China

**Keywords:** Developmental biology, Genetics, Medical research

## Abstract

To evaluate the correlation between chromosomal abnormalities and fetal aberrant right subclavian artery **(**ARSA) with or without additional ultrasound anomalies (UAs). A total of 340 fetuses diagnosed with ARSA by ultrasound between December, 2015, and July, 2021, were included. All cases were subdivided into three groups: (A) 121 (35.6%) cases with isolated ARSA, (B) 91 (26.8%) cases with soft markers, and (C) 128 (37.6%) cases complicated with other UAs. Invasive testing was performed via amniotic fluid or cord blood karyotyping and chromosomal microarray analysis (CMA) in parallel, and pregnancy outcomes were followed. Karyotype abnormalities were identified in 18/340 (5.3%) patients. Karyotype abnormalities in Groups A, B, and C were 0/121 (0.0%), 7/91 (7.7%), and 11/128 (8.6%), respectively. CMA abnormalities with clinically significant variants were detected in 37/340 (10.9%) cases, of which 22q11.2 deletion syndrome and trisomy 21 accounted for 48.6% (18/37). The overall abnormal CMA with clinically significant variant detection rates in Groups A, B, and C were 3/121(2.5%), 13/91 (14.3%), and 21/128 (16.4%), respectively. There were significant difference in clinically significant CMA anomalies detection rate between Groups A and C (*p* < 0.05), as well as Groups A and B (*p* < 0.05). Comparing CMA to karyotyping showed a clinically significant incremental yield in Group C (7.8%, 10/128) compared to Groups A (2.5%, 3/121) and B (6.6%, 6/91) (*p* > 0.05). Fetal ARSA with additional UAs, concurred with cardiac and extra-cardiac anomalies, constitutes a high-risk factor for chromosomal aberrations, especially for pathogenic or likely pathogenic copy number variants.

## Introduction

The aberrant right subclavian artery (ARSA), also defined as an aberrant retroesophageal right subclavian artery, is the most common aortic arch branching anomaly^1^. ARSA usually does not cause symptoms; however, newborns diagnosed with ARSA may have a variety of phenotypes such as dysphagia, respiratory distress, and stridor arising from esophageal and tracheal compression ^2–5^.

The prevalence of ARSA in normal individuals varies from 0.35% to 3.5% ^6^ , and 0.6–1.5% prenatally^7^. Isolated ARSA may not be clinically significant. However, it may indicate chromosomal abnormalities in the fetus, particularly trisomy 21^8^.

ARSA may be a normal variant in the general population or may be correlated with congenital heart defect (CHD) prenatally^9^ and different chromosomal abnormalities, especially trisomy 21 and 22q11 deletion syndrome compatible with DiGeorge syndrome (DGS)^10–12^. Studies have shown that patients with DGS often present with ARSA and a right aortic arch^13,14^. Given that ARSA and other vascular and cono-truncal defects are associated with DGS, isolated fetal ARSA may be an independent risk factor for DGS^15^. Thus, fetal ARSA requires serious attention and consideration.

Chromosomal microarray analysis (CMA) is considered a first-tier method for detecting microscopic and submicroscopic chromosomal abnormalities, and it yields a significantly higher detection rate of copy number variations (CNVs) than karyotyping. Limited evidence with a large population has been published describing the value of CMA in pregnancies with fetal ARSA^16^. The objective of the present study was to evaluate the prevalence of chromosomal aberrations in 340 fetal ARSA.

## Materials and methods

### Study population

This retrospective study reviewed 340 fetuses diagnosed with ARSA using ultrasound between December, 2015, and July, 2021, in a tertiary referral center. All pregnant women in this study conceived naturally. Conventional fetal karyotyping and CMA testing were performed concurrently in all fetuses. The specimens included 292 amniotic fluid samples and 48 umbilical cord blood samples. The most common indication for cordocentesis was fetal risk of severe thalassemia, rapid karyotyping, fetal suspected congenital infections (rubella /varicella), and oligohydramnios, etc. Demographic characteristics are summarized in Table 1. A total of 340 fetuses were classified into three groups: isolated ARSA (Group A), ARSA accompanied with soft markers (Group B), and ARSA accompanied with other ultrasound anomalies (Group C). Base on the recent guidelines^17,18^, the soft markers we have used in the study include echogenic bowel, pyelectasis, echogenic intracardiac focus, increased NT thickness, thick nuchal fold, nasal bone dysplasia, absence of nasal bone, EIF, mild ventriculomegaly, single umbilical artery, choroid plexus cysts, and cystic hygroma.

**Table 1:** Demographic characteristics of the 340 fetuses with ARSA.

Variant	Total (n = 340)	Group A(n = 121)	Group B(n = 91)	Group C(n = 128)
Maternal age (mean ± SD)	33.1 ± 2.5	34.3 ± 2.3	34.1 ± 2.0	32.2 ± 1.9
Gestation weeks at invasive PD (mean ± SD)	22.3 ± 3.1	23.2 ± 2.2	22.5 ± 1.5	24.5 ± 2.4
**Specimens**				
Amniotic fluid n (%)	292 (86.0%)	108 (89.2%)	77 (84.5%)	108 (84.1%)
Cord blood n (%)	48 (14.0%)	13 (10.8%)	14 (15.5%)	20(15.9%)
**Pregnancy outcome**				
CTP n (%)	293 (86.2%)	119(98.3%)	78 (85.7%)	96 (75.0%)
TOP/IUFD n (%)	47 (13.8%)	2(1.7%)	13 (14.3%)	32 (25.0%)

Group A = isolated ARSA; Group B = ARSA accompanied with soft ultrasound markers; Group C = ARSA accompanied with additional ultrasound malformations.

ARSA aberrant right subclavian artery, TOP termination of pregnancy, CTP continuation of pregnancy, IUFD intrauterine fetal demise, SD standard deviation, PD prenatal diagnosis.

Follow-up was performed via medical records or telephone calls, and clinical and imaging examinations were performed in born infants, ranging from three months to two years after birth. The study was approved by the Ethics Committee of Fujian Maternity and Child Health Hospital (No.2016KYLLD01051). All methods were carried out in accordance with relevant guidelines and regulations, and patients signed an informed consent form.

### Conventional karyotyping analysis

Karyotyping was performed following the standard procedures, and karyotypes were scanned on Leica GSL120. At least 20 metaphases were counted, and five metaphases were analyzed. Abnormal karyotypes were named basing on ISCN 2020.

### Extraction of genomic DNA and CMA

Genomic DNA from the fetus and its parents were extracted using the QIAamp® DNA Blood Mini Kit (Qiagen Inc., Hilden, Germany) according to the manufacturer’s instructions, and maternal cell contamination was ruled out using microsatellite DNA linkage analysis.

CMA was carried out using Affymetrix CytoScan 750 K array (Affymetrix Inc., Santa Clara, CA), and data was analyzed via Affymetrix Chromosome Analysis Suite Software (version 3.1.0.15) as previously described^19^. The reporting threshold was set at gains ≥ 1 Mb, losses ≥ 500 Kb and loss of heterozygosity (LOH) ≥ 10 Mb. For fetuses with abnormal CNVs, parental testing was performed to determine its origin. CNVs were classified through OMIM, UCSC, International Standard Cytogenomic Array, Database of Genome Variants, and Decipher databases into pathogenic, likely pathogenic (LP), variants of uncertain significance (VOUS), likely benign, and benign according to the American College of Medical Genetics guidelines^20^. Pathogenic/likely pathogenic CNVs were considered clinically significant. Parental microarray analysis was recommended to determine the origin of CNVs.

### Statistics

SPSS software version 19.0 (SPSS, Inc., Chicago, IL) was used for statistical analysis. Measurement data were expressed as mean ± standard deviation, statistical comparisons were performed using χ^2^ test, and *p* < 0.05 was considered statistically significant.

## Results

### Demographic characteristics of subjects

ARSA was diagnosed in 340 fetuses. Among them, 292 (86.0%) and 48 (14.0%) cases were detected by ultrasound in the 2nd, and 3rd trimester, respectively. Invasive procedures included 292 patients who underwent amniocentesis and 48 who underwent cord blood sampling. ARSA was an isolated finding in 121/340 cases (35.6%, Group A) and was accompanied by soft markers in 91/340 cases (26.8%, Group B), which was consistent with other UAs in the remaining 128/340 cases (37.6%, Group C). Additionally, 26 cases had only cardiac defects in Group C. The mean gestational age at the time of invasive testing was 23 weeks. A total of 303 (89.1%) pregnant women continued their pregnancies, 36 (10.6%) opted to terminate their pregnancies, and one (0.3%) woman whose fetus died in utero was followed up. The demographic characteristics of the 340 fetuses with ARSA are presented in Table 1.

### Abnormal karyotypes results

**Figure 1:**
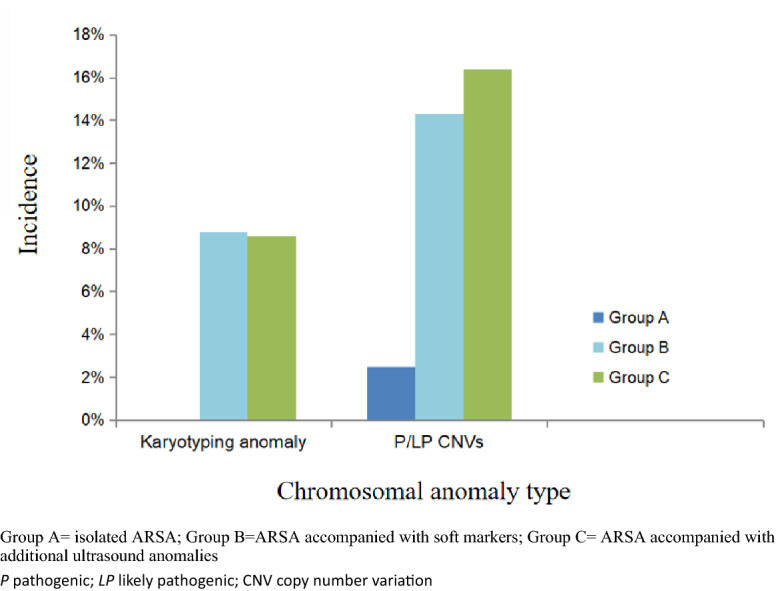
The incidence of chromosomal abnormalities in three subgroups. Group A = isolated ARSA; Group B = ARSA accompanied with soft markers; Group C = ARSA accompanied with additional ultrasound anomalies. *P* pathogenic; *LP* likely pathogenic; CNV copy number variation.

Among the 340 cases, karyotype abnormalities were identified in 18/340 (5.3%) cases, including trisomy 21 (n = 9) [including standard trisomy 21 (n = 7), 46,XX,der (15;21)(q10;q10)mat, + 21 (n = 1), and 46,XY,der(15;21)(q10;q10)mat,  + 21 (n = 1)], trisomy 18 (n = 3), turner syndrome(TS) (n = 2) [including 45,X (n = 1) and 45,X[16]/46,XY[8] (n = 1)], 46,XX,del(2)(q37) (n = 1), 46,XX,add(10)(q26) (n = 1), 46,XX,t(4;12)(p15.3;q13.1)pat (n = 1), and 45,XX,der(14;18)(q10;q10) pat (n = 1) (Table 2). Of the eighteen cases, trisomy 21 and trisomy 18 were the most common aneuploidies. Trisomy 21 was detected in nine cases (2.6%) (7 cases in Group B and 2 case in Group C) with additional UAs, all of which were terminated. Karyotype abnormalities were detected in 0/121 cases (0.0%) in Group A, 7/91 (7.7%) in Group B, and 11/128 (8.6%) in Group C. The frequency of clinically significant findings in Groups C and B was significantly higher than that in Group A. However, there was no significant difference between Groups B and C (7.7% vs 8.6%, *p* > 0.05) (Table 3 and Fig. 1).

**Table 2:** Characteristic of chromosomal abnormalities and CNVs detected by karyotyping and CMA in 48 fetuses diagnosed with ARSA.

Case number	Age (years)	Additional ultrasound findings (group)	Fetal CMA results (Size)	Fetal karyotype	Associated syndrome with invasive testing result	Parental origin of CMA results	Pathogenicity classification of CMA result	Pregnancy outcome
**Variants of clinical significance**								
1	28	None (Group A)	arr[GRCh37]22q11.21(18,648,856–21,915,207) × 3 (3.3 Mb)	46,XX	22q11.2 duplication syndrome	NA	P	CTP
2	23	None (Group A)	arr[GRCh37]22q11.21(18,631,364_21,800,471) × 1 (3.2 Mb)	46,XX	DGS	dn	P	TOP
3	29	None (Group A)	arr[GRCh37]4q24q25(107033067_109404131) × 1 (2.3 Mb)	46,XX	Non-syndromic	dn	LP	TOP
4	34	Left ventricular chordae tendinosus sound enhancement, echogenic bowel (Group B)	arr[GRCh37](21) × 3, 15q13.2q13.3(31,162,016_32,914,239) × 3 (1.7 Mb)	46,XX,der(15;21) (q10;q10)mat, + 21	Trisomy 21	mat	P VOUS	TOP
5	27	Bilateral mild hydronephrosis (Group B)	arr[GRCh37] 22q11.21(18,916,842_21,800,471) × 1 (2.9 Mb)	46,XY	DGS	dn	P	TOP
6	30	EIF (Group B)	arr[GRCh37]22q11.22q11.23(22,997,928_25,041,592) × 3 (2 Mb)	46,XX	22q11.2 duplication syndrome	mat	LP	TOP
7	30	Increased NT thickness, nasal bone dysplasia, EIF (Group B)	arr [GRCh37](21) × 3	47,XX, + 21	Trisomy 21	dn	P	TOP
8	36	Increased NT thickness (4.0 mm), nasal bone dysplasia, bilateral mild hydronephrosis, intestinal echo enhancement (Group B)	arr [GRCh37](21) × 3	47,XX, + 21	Trisomy 21	dn	P	TOP
9	27	Increased NT thickness (3.3 mm) (Group B)	arr[GRCh37]15q11.2q13.1(22770422_28928730) × 1(6.16 Mb)	46,XX	PWS/AS	dn	P	TOP
10	25	Cystic hygroma (Group B)	arrr [GRCh37] (X) × 1 ~ 2 CN: 1.8	46,XX	TS	dn	P	TOP
11	37	Increased NT thickness (4.3 mm), absence of nasal bone (Group B)	arr [GRCh37](21) × 3	47,XX, + 21	Trisomy 21	dn	P	TOP
12	36	VSD, mild tricuspid regurgitation, SUA, double superior vena cava (Group C)	arr[GRCh37]22q11.1q11.21(16,888,899_18,649,190) × 4 (1.7 Mb)	46,XY	Cat eye syndrome	dn	P	TOP
13	36	VSD,RAA, U-shaped vascular rings, AMA (Group C)	arr[GRCh37]22q11.21(18,648,855_21,800,471) × 1 (3.1 Mb)	46,XX	DGS	dn	P	TOP
14	36	RAA, mild tricuspid regurgitation (Group C)	arr[GRCh37]1q42.12q44(226,842,481_248,545,364) × 3 (21.7 Mb)	46,XX	Partial trisomy of 1q	dn	P	TOP
15	26	RAA, left ductus arteriosus (U-shaped vascular rings) (Group C)	arr[GRCh37]2q37.1q37.3(234,308,645–242,782,258) × 1 (8.4 Mb)	46,XX,del(2)(q37) dn	2q37 monomer syndrome	dn	P	TOP
16	31	Strephenopodia (Group C)	arr[GRCh37]17p12p11.2(15,759,453_20,547,625) × 3 (4.7 Mb)	46,XY	Potocki-Lupski syndrome	dn	P	TOP
17	24	FGR, thick nuchal fold, PLSVC (Group C)	arr[GRCh37]10q26.2q26.3(128,251,975_135,426,386) × 1 (7.1 Mb), 11q23.3q25(116,683,754_134,937,416) × 3 (18.2 Mb)	46,XX,add(10)(q26) dn	None	dn dn	P P	TOP
18	38	RAA, left ductus arteriosus, U-shaped vascular rings (Group C)	arr[GRCh37]22q11.21(18,649,189_20,312,661) × 3 (1.66 Mb)	46,XX	22q11.2 duplication syndrome	NA	LP	TOP
19	27	Bilateral pyelectasis (Group B)	arr[GRCh37]22q11.21(18,916,842_21,800,471) × 1 (2.9 Mb)	46,XY	DGS	dn	P	TOP
20	26	Hydramnios, EIF (Group C)	arr[GRCh37]22q11.21(18,916,842_21,800,471) × 1 (3.1 Mb)	46,XY	DGS	dn	P	TOP
21	26	VSD, right shift heart, right heart is bigger than left heart, pulmonary artery widening with little pulmonary valve regurgitation, echogenic bowel, high risk for trisomy 18 (Group C)	arr[GRCh37](18) × 3	47,XX, + 18	Trisomy 18	dn	P	TOP
22	29	Bilateral pyelectasis (Group B)	arr[GRCh37]22q11.21(18,636,749_21,800,471) × 1 (3.16 Mb)	46,XY	DGS	dn	P	TOP
23	37	Pericardial effusion, thick nuchal fold, nasal bone dysplasia, mild tricuspid regurgitation, (Group C)	arr[GRCh37](21) × 3	47,XY, + 21	Trisomy 21	dn	P	TOP
24	42	Nasal bone dysplasia, EIF (Group B)	arr [GRCh37](21) × 3	47,XX, + 21	Trisomy 21	dn	P	TOP
25	24	Increased NT thickness (4.3 mm), EIF, venous catheter α wave reverse (Group B)	arr [GRCh37](21) × 3	46,XY,der(14;21)(q10;q10) mat, + 21	Trisomy 21	mat	P	TOP
26	33	Truncus arteriosus A1 type, VSD (Group C)	arr[GRCh37]22q11.21(18631364_20312661) × 1 (1.68 Mb)	46,XX	DGS	dn	P	TOP
27	30	RAA (Group C)	arr[GRCh37]22q11.21(18636749_21800471) × 1(3.1 Mb)	46,XX	DGS	dn	P	TOP
28	37	VSD, coarctation of the aorta (Group C)	arr[GRCh37]22q11.21(18631364_21800471) × 1 (3.1 Mb)	46,XY	DGS	dn	P	TOP
29	35	Increased NT thickness (5.4 mm), tachycardia, neck hygroma (Group C)	arr[GRCh37]3p22.1p21.31(40512685_45189740) × 3, (4.7 Mb) 18p11.32p11.21(136228_15099116) × 1 (15 Mb)	45,XX, der(14;18) (q10;q10) dn	None 18p deletion syndrome	pat dn	VOUS P	TOP
30	47	Increased NT thickness (4.0 mm), nasal bone dysplasia, bilateral pyelectasis, echogenic bowel, (Group B)	arr[GRCh37] (21) × 3	47,XX, + 21	Trisomy 21	dn	P	TOP
31	22	Enlarged right atrium and right auricle, VSD, Blake's pouch cyst, thick nuchal fold (Group C)	arr[GRCh37]2q13(111,397,196_113,111,856) × 1 (1.7 Mb)	46,XX	Phelan–McDermid syndrome	mat	LP	TOP
32	31	Anasarca, nuchal cystic hygroma, severe tricuspid regurgitation, SUA, the a-wave notch of the blood spectrum of the venous catheter deepened (Group C)	arr[GRCh37] (18) × 3	47,XY, + 18	Trisomy 18	dn	P	TOP
33	38	Holoprosencephaly, cleft palate, venous catheter α wave reverse, echogenic bowel (Group C)	arr[GRCh37] (18) × 3	47,XY, + 18	Trisomy 18	dn	P	TOP
34	31	Fetal skin edema all over the body, increased NT thickness, bilateral pleural effusion, PLSVC, venous catheter α wave reverse (Group C)	arr r[GRCh37](X) × 1	45,X	TS	dn	P	TOP
35	41	VSD (Group C)	arr [GRCh37](21) × 3	47,XY, + 21	Trisomy 21	dn	P	TOP
36	33	Left aortic arch (Group C)	arr[GRCh37] 17p12(14087919_15413862) × 3 (1.3 Mb)	46,XX	Charcot-Marie-Tooth 1A type (CMT1A), including *PMP22*	dn	P	TOP
37	30	Cysts at the cisterna of the tetrad, mild tricuspid regurgitation (Group C)	arr[GRCh37] Yp11.32 q11.223 (118552_24890379) × 2, (24.7 Mb) Yq11.223q11.23(24985376_28799654) × 0 (3.8 Mb)	45,X^16^/46,XY^8^	TS	dn	P	TOP
**Variants of non-clinical significance**								
38	32	None (Group A)	arr[GRCh37]10q21.3(68437064_68686435) × 1 (0.25 Mb)	46,XY	None	NA	VOUS	Live birth (normal)
39	25	None (Group A)	arr[GRCh37]16p11.2(28,786,703_29,032,280) × 3 mat (0.25 Mb)	46,XX	None	mat	VOUS	Live birth (normal)
40	18	None (Group A)	arr[GRCh37]7q34(139,340,641_139,769,640) × 3 mat (0.43 Mb)	46,XX	None	mat	VOUS	Live birth (normal)
41	25	None (Group A)	arr[GRCh37]16p11.2(28,786,703_29,032,280) × 3 mat (0.25 Mb)	46,XX	None	dn	VOUS	CTP
42	28	Bilateral pyelectasis, echogenic bowel (GroupB)	arr[GRCh37] 18p11.31p11.23(6823577_8167871) × 3 (1.3 Mb)	46,XY	None	pat	VOUS	CTP
43	31	Hydramnios (Group C)	arr[GRCh37]1p36.21p35.2(15,728,288_31,781,279) × 2 hmz, (16 Mb) 4p15.2p11(25,981,952_49,063,479) × 2 hmz (23 Mb)	46,XX	None None	dn	VOUS VOUS	CTP
44	27	Severe FGR, thick nuchal fold, prefrontal skin thickened, smaller ears, small left heart, small inner diameter of the aorta, SUA, echogenic bowel, abnormal connection of the venous catheter, echo enhancement in both renal parenchyma (Group C)	arr[GRCh37]16q22.2q23.2(71463698_79614082) × 3 (8.15 Mb)	46,XX	None	NA	VOUS	IUFD
45	21	Right strephenopodia (Group C)	arr[GRCh37]Xp22.31(6460001_8210000) × 3 (1.75 Mb)	46,XX	None	NA	VOUS	Live birth (normal)
46	26	Intestinal duplication? (Group C)	arr[GRCh37](1–22, X) × 2	46,XX,t(4;12) (p15.3;q13.1) pat	None	pat		CTP
47	29	RAA, left ductus arteriosus (U-shaped vascular rings) (Group C)	arr[GRCh37]10q22.3q23.2(81,598,041_88,975,507) × 3 (7.3 Mb)	46,XY	None	NA	VOUS	CTP
48	29	RAA, left ductus arteriosus (U-shaped vascular rings) (Group C)	arr[GRCh37]10q22.3q23.2(81,598,041_88,975,507) × 3 (0.73 Mb)	46,XY	None	dn	VOUS	CTP

*CMA* chromosomal microarray analysis, *DGS* DiGeorge syndrome, *FGR* fetal growth restriction, *RAA* right aortic arch, VOUS variants of uncertain significance, *VSD* ventricular septal defect*, CTP* continuation of pregnancy, *TOP* termination of pregnancy, *N*A not available, *SUA* single umbilical artery, *P* pathogenic, *LP* likely pathogenic, *mat* maternal, *pat* paternal, dn, de novo, *EIF* echogenic intracardiac focus, *NT* nuchal translucency, *IUFD* in utero fetal death, *PLSVC* persistent left superior vena cava, *TS* turner syndrome.

**Table 3:** Distribution of chromosomal aberration findings in fetal ARSA with or without other UAs.

ARSA classification	Group A (n = 121)	Group B (n = 91)	Group C					Total (n = 340)
			Cardiac defects (n = 26)	Extracardiac defects (n = 18)	Cardiac + extracardiac defects (n = 6)	Other UAs (n = 78)	Total (n = 128)	
Abnormal karyotypes (n,%)	0 (0.0%)	7 (7.7%)	2(7.7%)	4(22.2%)	1(16.7%)	4(5.1%)	11(8.6%)	18 (5.3%)
Clinically significant CMA result (n,%)	3 (2.5%)	13 (14.3%)	6(23.1%)	4(22.2%)	2(33.3%)	9(11.5%)	21(16.4%)	37 (10.9%)
pCNVs	2 (1.7%)	12 (13.2%)	6(23.1%)	4(22.2%)	1(16.7%)	8(10.3%)	19(14.8%)	33 (9.7%)
Likely pCNVs	1 (0.8%)	1 (1.1%)	0(0.0%)	0(0.0%)	1(16.7%)	1(1.3%)	2(1.6%)	4 (1.2%)

Group A = isolated ARSA; Group B = ARSA accompanied with soft markers; Group C = ARSA accompanied with additional ultrasound anomalies.

*ARSA* aberrant right subclavian artery, *CMA* chromosomal microarray analysis, *UA* ultrasound anomaly, *p* pathogenic.

The incidence of abnormal karyotypes in the advanced maternal age (AMA) and the young maternal age (YMA) group was 15.1% (8/53), and 3.5% (10/287), respectively. There is significant difference in the incidence rate of abnormal karyotypes between the two groups (*p* < 0.05). More information is displayed in Table 4.

**Table 4:** Distribution of chromosomal aberration findings in fetal ARSA combined with AMA and YMA.

Maternal age	Abnormal karyotype (n, %)				Clinically significant microarray results (n, %)
	T21	T18	Other aneuploidy	Structual anomaly	
AMA (n = 53)	6 (11.3)	1 (1.9)	0 (0)	1 (1.9)	13 (24.5)
YMA (n = 287)	3 (1.0)	2 (0.7)	2 (0.7)	3 (1.0)	24 (8.4)

*AMA* advanced maternal age, *YMA* young maternal age,* T* trisomy, *ARSA* aberrant right subclavian artery.

### Abnormal CMA results

Abnormal CMA results were identified in 48 cases (14.1%), including thirty-three cases (9.7%) of pathogenic CNVs, four cases (1.2%) of LP CNVs, and eleven cases (3.2%) of VOUS, with overall CMA anomaly detection rates of 5.8% (7/121), 16.5% (15/91), and 20.3% (26/128) in Groups A, B, and C, respectively. The clinically significant CMA anomalies detection rate in Groups A, B, and C were 2.5% (3/121), 14.3% (13/91), and 16.4% (21/128), respectively, and the details are summarized in Tables 2 and 3. Submicroscopic CNVs of clinical significance (*n* = 25) ranged in size from 166 Kb to 21.7 Mb, and 20 of them were associated with the following clinical syndromes: DGS (OMIM # 611,867, cases 2, 5, 13, 19, 20, 22, and 26–28), 22q11.2 duplication syndrome (OMIM # 608,363, case 1, 6 and 18), Potocki-Lupski syndrome (OMIM # 610,883, case 16), Phelan-McDermid syndrome (OMIM # 606,232, case 31), cat eye syndrome (OMIM # 115,470, case 12), 2q37 monomer syndrome (case 15), partial trisomy of 1q (case 14), prader-willi syndrome (PWS)/angelman syndrome (AS) (case 9), Charcot-Marie-Tooth 1A type (CMT1A) (case 36), and 18p deletion syndrome (case 29). 22q11.2 deletion, compatible with DGS and trisomy 21, accounted for 48.6% (18/37) of the clinically significant variants. Variants derived from 22q11.2, comprising nine cases of deletion and three cases of duplication, were observed with the highest frequency among the variants of clinical significance (32.4%, 12/37). In addition, CMA yielded one case of LOH involving 1p36.21p35.2 and 4p15.2p11 from one fetus with ARSA combined with hydramnios (case 42). The incremental yield of clinically significant findings in Group C (7.8%, 10/128) was much higher than that in Groups A (2.5%, 3/121) and B (6.6%, 6/91) (7.8% vs. 6.6% vs. 2.5%, *p* > 0.05), when compared with karyotyping.

In Group A, the rate of clinically significant CNVs detected by CMA was 2.5% (3/121). We found three fetuses (Table 2, case 1, 2 and 3), one each of with DGS, 22q11.2 duplication syndrome, and deletion in 4q24q25 in whom the only ultrasound anomaly was an ARSA. In Group B, eight cases of clinically significant CMA findings were noted (Table 2, cases 4–11). In Group C, 28 clinical significance CNVs were identified in 26 fetuses via CMA (Table 2, cases 12–37). It is worth noting that 73 fetuses presented with additional cardiac anomalies, 20 cases presented with extra-cardiac anomalies, and 35 cases presented with both cardiac and extra-cardiac anomalies, and the frequencies of clinically significant CNVs findings by CMA showed no significant difference (12.3% vs. 20.0% vs. 22.9%, *p* > 0.05).

In our data set, the incidence of clinically significant microarray results in the AMA and YMA group was 24.5% (13/53) and 8.4% (24/287), respectively. There is significant difference in the incidence rate of clinically significant microarray results between the two groups (*p* < 0.05) (Table 4).

In addition, structural rearrangements account for 33.3% (6/18) of all abnormal karyotypes. After blood cytogenetic analyses of the couples to assess the origin of the structural rearrangement, three cases were found to be inherited from a parental translocation and three cases appeared de novo.

### Prenatal associated features in fetal ARSA

In Group A, 121 of the ARSA cases (35.6%) were isolated, whereas in Group B, 91 of the ARSA cases were accompanied by soft markers, of which the most common top three soft ultrasound markers were echogenic intracardiac focus (EIF) (33/91, 36.3%), followed by heart valve regurgitation (33.0%, 30/91) and pyelectasis (20.9%, 19/91)). In Group C, 128 fetuses had additional ultrasound anomalies (UAs); ARSA was accompanied by only cardiac defects in 26 cases (20.3%), extracardiac anomalies in 18 cases (14.1%), both cardiac and extracardiac anomalies were present in 6 cases (4.7%), and the other UAs were present in 78 cases, among which the most frequent anomalies were right aortic arch (RAA) (35.9%, 46/128), followed by left ductus arteriosus (25.8%, 33/128), VSD (21.1%, 27/128), cranial system anomalies (10.2%, 13/128), fetal urinary system anomalies (9.4%, 12/128), and fetal growth restriction (7.0%, 9/128).

### Follow-up of pregnancy outcome

Among all fetuses with ARSA, 186 were male and 154 were female (54.7% vs. 45.3%). Follow-up was performed for all patients (100%). Pregnant women whose fetuses had normal karyotype and CMA results continued with their pregnancies, except for ten fetuses with multiple congenital anomalies (MCA). Overall, forty-seven (13.8%) fetuses were terminated, including two fetuses in Group A, thirteen fetuses in Group B, and thirty-two fetuses in Group C. One fetus carrying the 16q22.2q23.2 duplication with MCA died in utero (case 44). All infants underwent regular physical examinations, and no neonatal complications resulting from esophageal and tracheal compression at birth were found. However, one male infant at three months after birth (Group A), nystagmus, was unable to chase light, accompanied with strabismus. Three patients with cardiac and extracardiac defects underwent surgical procedures; Echocardiography was performed postnatally in only 31 cases after birth, among them, a small partial atrioventricular septal defect and hypospadias were detected postnatally on echocardiography in one case, the rest of the infants showed normal development postnatally.

## Discussion

Embryonic development of the aorta occurs during the 3^rd^ gestation age^21^. Two symmetric aortic arches form a vascular ring, around the trachea and esophagus, connecting the ascending and descending aortas^22^. Each aortic arch gives rise to a common carotid artery and a subclavian artery. On each side, right- and left-sided ducti arteriosi, connecting the pulmonary arteries to the distal part of each aortic arch, form an additional vascular ring^23^. Typically, the aortic arch branches into three vessels: the brachiocephalic trunk, the left subclavian artery and the left common carotid artery^24^.

Aortic arch anomalies refer to the position or branching pattern anomalies of arcus^25^. Other structural (cardiac defects) or chromosomal or genetic anomalies can be triggered by aortic arch anomalies^26,27^. ARSA is one of the aortic arch branching malformations^23^. In this ARSA situation, there are four arteries on the left aortic: the left common carotid, the left subclavian, the right common carotid, and the ARSA^28^.

The ultrasonic examination of aortic arch anomalies lies in the 3-vessel and trachea (3VT) view and the subclavian artery view^23,29^, basing on the cardiovascular system sonographic evaluation protocol^30^. Furthermore, the use of colour Doppler improves the accuracy of the visualization and understanding of the 3VT view. The ultrasound 3VT view shows the normal anatomic appearance of the great vessels: the left-sided ductus arteriosus and the transverse portion of the aortic arch forming a V-shaped structure on the left of the trachea and a transverse section of the superior vena cava. When moving to the subclavian artery view, the normal handlebar positioning of the subclavian arteries is absent, and the arteries appear straight.

The ARSA is a variation, where the RSA arises directly from the aortic arch, crosses to the right side behind the trachea and the esophagus and turns toward the right shoulder. ARSA may either be a normal variant in general population or it can be associated with chromosomal abnormalities and cardiac defects^31^.

Studies have shown that isolated ARSA is a soft marker for trisomy 21^1^. Both Paladini et al.^32^ and Borenstein et al.^8^ found fetuses with isolated ARSA carrying trisomy 21. However, whether fetal karyotype analysis should be offered when identifying isolated ARSA is still controversial^11,16^, especially regarding the association between isolated ARSA and trisomy 21^1,7,12,32–34^. In our series, trisomy was not detected in 121 fetuses with isolated ARSA. Thus, an isolated ARSA may not be a strong independent indicator of trisomy. The conflicting data were mainly due to the combination of other high-risk pregnancies, such as high-risk for trisomy 21, advanced maternal age, and different sample sizes.

The positive likelihood ratio in cases of non-isolated ARSA was 26.81 for trisomy 21^11^. Several studies have reported a positive association between non-isolated ARSA and trisomy 21^11,35,36^. In the present study, we found a low prevalence (5.0%, 11/219) of trisomy (eight trisomy 21 and three trisomy 18) in fetuses diagnosed with non-isolated ARSA but none in the 121 fetuses with isolated ARSA, which is consistent with the reported studies on the positive correlation between non-isolated ARSA and trisomy 21; however, the incidence is significantly lower than that (35.7%) reported by Svirsky R, et al. ^15^. This difference may be due to different sample sizes and study population.

De León-Luis et al.^12^ showed that there is no association between isolated ARSA and trisomy 21; thus, we differentiated it from isolated ARSA with additional UAs in our study. In recent studies, no cases of trisomy 21 or pathogenic CNVs (pCNVs) have been reported in fetuses with isolated ARSA^10–12,15^. However, in the present study, no cases of trisomy, but 3 fetuses (case 1–3) with pCNVs, were detected in 121 fetuses with isolated ARSA. In case 3, the finding of the 4q24q25 microdeletion in Group A was almost certainly a coincidence because this variant is associated with ectodermal dysplasia, not with ARSA^37^. Our results contradict the data of Maya et al.^[11]^who found no pCNVs among 36 fetal isolated ARSA**,** which may be due to the different sample sizes. Scala et al.^1^ showed that ARSA is a clinically important soft marker of trisomy 21, but not sufficient to recommend fetal karyotyping in fetal isolated ARSA. Our finding supports this view of point.

All abnormal karyotypes except case 46 [the karyotype of the fetus (case 46) was 46,XX,t(4;12)(p15.3;q13.1)] were detected by CMA. Overall, the chromosomal findings in 19 of the 37 cases with P/LP CNVs in our cohort would not have been detected by traditional cytogenetics analysis. Therefore, CMA is recommended as a first-line detection method for chromosomal submicroscopic aberrations in fetuses diagnosed with ARSA.

Additional UAs were present in 219 fetuses, 34 (15.5%) of which had pathogenic CMA results. Among them, 22q11.2 microdeletion/microduplication was the most frequent variation, with nine cases of microdeletion and three cases of microduplication. The 22q11.2 microdeletion accounted for 42.9% (9/21) of fetuses with clinically significant submicroscopic chromosomal abnormalities. Some studies have shown that the risk is significantly increased when extra-cardiac malformations (especially thymus and parathyroid dysplasia) existed^38^. In our study, one fetus (case 5) with 22q11.2 deletion were identified in group B, and seven fetuses (case 13, 19, 20, 22, 26–28) with DGS were detected in group C. Our findings suggest that fetuses with non-isolated ARSA had a higher incidence (15.5%) of clinically significant CMA results than that in fetuses (2.5%) with isolated ARSA. Based on our data and published literature, when ARSA is detected prenatally by ultrasound, a detailed fetal anatomy scan is essential to determine whether ARSA is combined with additional UAs. If other UAs are found, invasive diagnostic procedures should be performed for microarray analysis to exclude fetal chromosomal aberrations. However, data on the association between ARSA and chromosomal abnormalities are conflicting, possibly because some earlier studies did not clearly distinguish between isolated and non-isolated ARSA^8^.

Previous studies have suggested that trisomy 21 and 22q11.2 microdeletion are the most associated chromosomal aberrations in fetal ARSA^10,11,39^. In our series, the 22q11.2 deletion (nine cases) compatible with DGS and trisomy 21 (nine cases) accounted for 48.6% (18/37) of the clinically significant variant, which is consistent with the study reported by Maya et al.^11^.

Nearly 90% of cases with DGS result came from a common 3 Mb microdeletion, 7% have an approximate 1.5 Mb microdeletion, and the remaining have a smaller deletion in the same region^40^. In our series, all fetuses diagnosed with DGS carry a frequent 3 Mb microdeletion except in case 26. Patients with the DGS presented variable phenotypes^13^. Cardiac defects are the most common abnormality (80%), especially in conotruncal cardiac defects such as aortic arch interruption, tetralogy of fallot, and complete transposition of the great arteries^41^. Additionally, individuals with DGS frequently have vascular anomalies such as a RAA and ARSA^14^. In our cohort, totally, seven fetuses (Cases 13, 19, 20, 22, 26–28) with DGS were identified in Group C, of which, two cases (28.6%) have a RAA and ARSA (Table 2).

De Leon-Luis´et al*.*^12^ reported on a fetus with ARSA accompanied by hypoplastic left ventricle who carried a 22q11.2 microduplication. In our cohort, another CNV that occurred with high frequency was 22q11.2 microduplication, which accounted for 8.1% (3/37) of fetuses with clinically significant CMA results; of these, one case with RAA, left ductus arteriosus, and U-shaped vascular rings was detected in Group C (case 18), another case with EIF was reported in Group B (case 6) , and the other case was reported in Group A (case 1). Affected individuals with 22q11.2 duplication syndrome are at increased risk for a variety of problems including gastrointestinal complications, endocrine dysfunction, ophthalmologic abnormalities, palatal anomalies, CHD, musculoskeletal differences, and neurologic abnormalities^42^. Given the UAs and genetic abnormalities, labor was induced in cases 6 and 18, whereas fetus in case 1 was termed delivery with normal development at 1.5 years.

In the present study, of the included 340 cases, an additional cardiac defects were present in 32 cases (9.4%), which is slightly lower than that (10.7%) reported by Song et al.^43^, this is mainly due to the different sample size and study population, the cardiac malformations include pulmonary atresia, right displacement of heart, persistent left superior vena cava, pulmonary artery stenosis, small left heart, aortic straddle, ventricular septal defect, and tricuspid atresia. Additionally, the detection rate of extra-cardiac anomalies with ARSA has been reported in ~ 5%-26.7% of cases^12^. Twenty cases (5.9%) of ARSA were accompanied with only extra-cardiac anomalies, which is in agreement with the reported researches^12^.

Some scholars have even concluded that CMA had no additive value in such cases^16^. The conflicting evidence in the literature regarding the association of ARSA and chromosomal abnormalities is probably because earlier studies did not differentiate between isolated ARSA and non-isolated ARSA. However, detailed ultrasound screening should be performed to confirm the presence of coexisting malformations. In Group C, thirty-five cases had cardiac and extra-cardiac anomalies, and the incidence of clinically significant CMA result was as high as 22.9% (8/35), which further highlights the importance of a detailed ultrasound scan for additional anomalies when ARSA is encountered, and fetal CMA is recommended.

With the increasing maternal age, AMA would become more prone to nondisjunction due to age-related meiotic errors in oogenesis^44^. It is well known that AMA is associated with an increased risk for fetal Down syndrome. An AMA was significantly associated with the incidence of chromosomal abnormality, particularly autosomal trisomies^45^. In addition, Chen LP, et al.^46^ showed that with combined ARSA and AMA, the likelihood of the incidence of abnormal karyotype increased, and the frequency of abnormal karyotype was much higher in the AMA group than that in the YMA Group.

In our data set, the incidence of abnormal karyotypes and clinically significant microarray results in the AMA group was 15.1% (8/53) and 24.5% (13/53), respectively, and in the YMA group, the incidence was 3.5% (10/287) and 8.4% (24/287), respectively. There is significant difference in the incidence rate of abnormal karyotypes and clinically significant microarray results between the two groups (*p* < 0.05), our data also confirm the point^46^.

For a previous fetus with trisomy 21 occurring by nondisjunction, invasive testing is recommended in the subsequent pregnancies. In our conhort, for the only one woman referred because of a family history of previous trisomy 21 de novo, fortunately, the fetus in this pregnancy has a normal karyotype.

In the present study, structural rearrangements account for 33.3% (6/18) of all abnormal karyotypes. Aminocentesis is also recommended for a pregnant couple with a structural balanced rearrangement, since the fetus could inherit an unbalanced rearrangement resulting in global developmental delay and other anomalies^47^. The risk estimated is variable as it depends on the breakpoints of each translocation^48^.

ARSA appears to have a female predominance^2,43,49,50^. In contrast, Zapata et al. ^51^ revealed an equal gender distribution of ARSA. Among all fetuses with ARSA in our study, 154 were female, and 186 were male (45.3% vs. 54.7%), males shows a higher prevalence of ARSA than in females, thus, a larger population study in multiple centers is needed.

Our study had several limitations. First, the study was retrospective in nature and the sample size was limited. Second, not all fetal ARSA were confirmed by imaging after birth; for asymptomatic infants, echocardiography is rarely routinely conducted postnatally, and thus the true prevalence of ARSA is underestimated. Third, chromosomal testing cannot identify single- gene diseases associated with ARSA.

In conclusion, the presence of isolated ARSA rarely correlates with chromosomal abnormality. CMA increases the diagnostic yield of clinically significant submicroscopic CNVs in fetuses with ARSA compared with karyotyping, especially in fetuses with additional UAs, thus, a detailed ultrasound scan, especially fetal echocardiography examination for additional UAs, should be conducted. Invasive diagnostic procedures should be performed for fetal microarray analysis as ARSA accompanying with other UAs, as non-isolated ARSA will confer a higher risk factor for chromosomal aberration.

## Data Availability

The datasets used and/or analyzed during the current study are available from the corresponding author on reasonable request.
